# Physical Activity and Cumulative Long-Term Care Cost among Older Japanese Adults: A Prospective Study in JAGES

**DOI:** 10.3390/ijerph18095004

**Published:** 2021-05-09

**Authors:** Hiroshi Hirai, Masashige Saito, Naoki Kondo, Katsunori Kondo, Toshiyuki Ojima

**Affiliations:** 1 Department of Regional Social Management, Faculty of Life and Environmental Sciences, University of Yamanashi, 4-4-37 Takeda, Kofu-shi, Yamanashi 400-8510, Japan; 2 Faculty of Social Welfare, Nihon Fukushi University, Okuda, Mihama-cho, Chita-gun, Aichi 470-3295, Japan; masa-s@n-fukushi.ac.jp; 3 Center for Well-Being and Society, Nihon Fukushi University, 5-22-35 Chiyoda, Naka-ku, Nagoya 460-0012, Japan; kkondo@chiba-u.jp; 4 Department of Social Epidemiology, Graduate School of Medicine and School of Public Health, Kyoto University, Yoshida-Konoe-cho, Sakyo-ku, Kyoto 606-8501, Japan; kondo.naoki.0s@kyoto-u.ac.jp; 5 Center for Preventive Medical Sciences, Department of Social Preventive Medical Sciences, Chiba University, 1-8-1 Inohana, Chuo-ku, Chiba-shi, Chiba 260-8670, Japan; 6 Department of Gerontological Evaluation, Center for Gerontology and Social Science, National Center for Geriatrics and Gerontology, 7-430 Morioka-cho, Obu, Aichi 474-8511, Japan; 7 Department of Community Health and Preventive Medicine, Hamamatsu University School of Medicine, 1-20-1 Handayama, Higashi-ku, Hamamatsu, Shizuoka 431-3192, Japan; ojima@hama-med.ac.jp

**Keywords:** physical activity, older adults, care cost

## Abstract

This study aimed to determine the impact of physical activity on the cumulative cost of long-term care insurance (LTCI) services in a cohort of community-dwelling people (65 years and older) in Japan. Using cohort data from the Japan Gerontological Evaluation Study (JAGES) on those who were functionally independent as of 2010/11, we examined differences in the cumulative cost of LTCI services by physical activity. We followed 38,875 participants with LTCI service costs for 59 months. Physical activity was assessed by the frequency of going out and time spent walking. We adopted a generalized linear model with gamma distribution and log-link function, and a classical linear regression with multiple imputation. The cumulative LTCI costs significantly decreased with the frequency of going out and the time spent walking after adjustment for baseline covariates. LTCI’s cumulative cost for those who went out once a week or less was USD 600 higher than those who went out almost daily. Furthermore, costs for those who walked for less than 30 min were USD 900 higher than those who walked for more than 60 min. Physical activity among older individuals can reduce LTCI costs, which could provide a rationale for expenditure intervention programs that promote physical activity.

## 1. Introduction

Many developed and developing countries are expecting to face a large increase in the proportion of older adults. Aging is associated with a higher risk of chronic diseases and functional and cognitive impairment [1,2]. As society ages, the need for medical and long-term care will increase due to the increasing number of elderly people requiring care and the lengthening of the care period. Consequently, the aging of the population will increase the cost of healthcare services.

Considerable evidence has shown that physical activity is positively associated with better overall health outcomes [3,4]. Physical activity reduces the risk of mortality [5,6], cardiovascular disease [7], diabetes mellitus [8], hypertension [9], obesity [10], glycemic control [11], pain and disability [12], bone and joint diseases [13], musculoskeletal diseases [14], cognitive and physical decline [15,16], and depression [17].

In Japan, it is reported that the national health care expenditure for the 2018 fiscal year was USD 382 billion (JPY 40 trillion), of which 24.4% was used for cardiovascular disease and 8.8% was used for diseases of the musculoskeletal system and connective tissue [18]. Disease categories related to physical inactivity account for 1/3 of all medical services spending for people aged 65 and older. Many studies have investigated the effects of physical activity on medical expenditure [19,20,21,22]. In Japan, the Ohsaki National Health Insurance (NHI) cohort study reported that medical expenditure per month was significantly reduced among those who spent a longer time walking [23,24]. Encouraging physical activity can be an investment in reducing not only medical costs but also long-term care costs. However, the actual magnitude of these costs saved by physical activity is unknown. Estimates of the costs of physical inactivity are useful for evaluating not only the benefits of healthcare interventions, but also cross-sector benefits in the healthcare sector resulting from investment in the non-healthcare sector. This study aimed to determine the impact of physical activity on the cumulative cost of long-term care insurance (LTCI) services.

## 2. Materials and Methods

### 2.1. Participants

Our data were derived from the Japan Gerontological Evaluation Study (JAGES), a large-scale cohort study of community-dwelling people aged 65 years or older, with no physical or cognitive disabilities and who were not receiving LTCI. The JAGES was initiated in 1999 to obtain scientific findings to serve as a basis for elderly care policies. The project has been extended to 41 municipalities in 19 prefectures and involved 200,000 participants as of 2016. The JAGES protocol was approved by the Ethics Committee on the Research of Human Subjects at Nihon Fukushi University (no. 10-05), details provided separately [25]. The JAGES questionnaire included items on comprehensive health and sociodemographic information at baseline, which allowed us to adjust for various potential confounders.

The data for Wave 3 of the JAGES were collected mostly through self-administered questionnaires, mailed to a random sample of functionally independent individuals, aged 65 years or older, living in the participating municipalities from August 2010 to December 2011. In the present study, we used data from 11 municipalities among all participating municipalities because the necessary outcome information was readily available only in these municipalities (response rate = 64.0%; 47,510/74,253). All community-dwelling people aged 65 and older without disability were study subjects in 8 municipalities, and systematic sampling was used for 3 municipalities with large populations. Of the 47,510 individuals who responded, 4490 were excluded from the analyses due to missing information on age, sex, or ID for data linkage. An additional 2055 participants were excluded because their activities of daily living were dependent. Furthermore, we excluded 1510 who died or were certified for LTCI benefits within 1 year from baseline, and 580 who moved out of the municipalities within the follow-up period. Therefore, the sample size in the present study was 38,875.

### 2.2. Measures

#### 2.2.1. Outcome Variable

The outcome variable was the cumulative cost of LTCI services during the follow-up period. The JAGES collected information about the cost of LTCI or deaths from the municipalities that were also LTCI insurers. We obtained the long-term care costs of insured services across 59 points every month for 5 years. Around 85% of participants were not certified as requiring long-term care in the follow-up period; therefore, around 85% of cumulative LTCI costs are zero. We added 0.5 to all values of cumulative LTCI costs. Since long-term care insurance (LTCI) was launched in Japan in FY2000, the method of certification for long-term care has been changed three times. The questionnaire for eligibility assessment was changed in FY2003, FY2006, and FY2009, and care level classification changed in 2006. The method of certification for long-term care has not changed after FY2009. This study started follow-up in 2010 and outcome data were not affected by these changes. We used a currency exchange rate of JPY 100 to USD 1.

#### 2.2.2. Explanatory Variables

The frequency of going out and time spent walking were the two explanatory variables employed. Concerning the assessment of the frequency of going outdoors, subjects were asked the question, “How often do you usually go outside the house?” Following previous research [26], the frequency was categorized as follows: almost every day; 2 or 3 times per week; or once a week or less. The question regarding walking time for each subject was worded as “How long do you walk a day, on average?” The time spent walking was categorized as >60 min, 30–60 min, and less than 30 min. A previous study reported the validity of a single-item questionnaire on walking among the Japanese population, using pedometer counts as the reference standard [27].

#### 2.2.3. Covariates

We considered possible confounding factors from the respondents’ demographic, socioeconomic, and health status, age, sex, marital status (married, divorced, widowed, never married), educational attainment (<6, 6–9, 10–12, ≥13 years of schooling), household equivalized income (USD <20, 20–40, and ≥40), chronic medical conditions (stroke, heart disease, diabetes mellitus, arthritis, fracture, osteoporosis, visual impairment, hearing impairment, urination disorders), physical function (climbing stairs, rising from chair), self-rated health (very good, good, fair, or poor), and the 15-item Geriatric Depression Scale (0–4 no depression, 5–10 mild depression, and 11–15 severe depression). We used the fractional polynomials method to select powers for age. We selected age cubed divided by 1000 and used it in analysis as an age variable. Missing values in the covariates were dummy coded and included as the “Missing” category in analysis.

### 2.3. Statistical Analysis

A generalized linear model (GLM) with Tweedie distribution and log-link function, with a robust estimation of variance components, was used to estimate the dependent variables’ predicted value because the cumulative cost of LTCI services presents highly skewed, heavy-tailed distributions. For comparison, we performed ordinary least squares (OLS) regression with logarithmic transformation. The primary analysis was performed as a complete case analysis, with sensitivity analyses using multiple missing data imputations. We employed numerous imputation techniques by chained equations under the missing at random assumption to impute the missing values in the explanatory variables. We created 20 imputed datasets. Using each dataset, we recomputed the predicted value of the dependent variable by GLM and OLS. Finally, we calculated the adjusted mean of the estimated cumulative cost of LTCI services by physical activity, using GLM. We performed analyses using IBM SPSS Statistics 25 (IBM Corp., Armonk, NY, USA).

### 2.4. Patient and Public Involvement

No patient was involved in developing the research questions, outcome measurements, or design of the study. The results of this research will be disseminated to stakeholders, including local and central health governments, after being published in a scientific journal.

## 3. Results

Table 1 shows the characteristics of the respondents and the average cumulative LTCI cost. The mean age at baseline was 73.6, and 53.3% of the respondents were women.

Table 2 shows the certification rate as requiring long-term care and mortality for participants by grouping of the frequency of going out and the time spent walking.

The physically inactive group had a higher rate of those certified as requiring long-term care and mortality than the physically active group. Among all the participants, 5737 had functional limitations, as verified by their certification for LTCI benefits during the follow-up period, which included 2302 participants who went out almost every day (25.5/1000 person-years), 1787 who went out two or three times per week (23.3/1000 person-years), and 1246 who went out once a week or less (50.7/1000 person-years).

Table 3 and Table 4 show the results of the GLM and OLS analyses for the cumulative LTCI cost.

The frequency of going out showed a consistent negative association with the cumulative costs of LTCI. These cumulative costs were significantly higher in those who went out “once a week or less” and “2 or 3 times per week” than in those who went out almost every day.

The time spent walking was also negatively associated with the cumulative costs of LTCI. These cumulative costs were significantly higher in those who walked for less than 30 min than in those who walked for more than 60 min. When comparing “30 to 60 min” and “more than 60 min”, statistically significant differences were observed only in the OLS analysis.

Table 5 shows the adjusted means of the cumulative LTCI costs by the frequency of going out and the time spent walking.

Cumulative LTCI costs were USD 3000 (95%CI: 2200–4100) in those who went out once a week or less, USD 2700 (95%CI: 2000–3700) in those who went out 2 or 3 times per week, and USD 2300 (95%CI: 1700–3100) in those who went out almost every day, after adjusting for age, sex, marital status, educational attainment, household equivalized income, chronic medical conditions, physical function, self-rated health, and depression by GLM analysis. Likewise, cumulative LTCI costs were USD 3200 (95%CI: 2200–4600) for those who walked for less than 30 min, USD 2400 (95%CI: 1700–3400) for those who walked for 30 to 60 min, and USD 2100 (95%CI: 1400–3000) for those who walked for more than 60 min. A sensitivity analysis using multiple imputations provided similar results to the complete case analysis.

## 4. Discussion

The physically inactive group had a higher certification rate for requiring long-term care. Regarding frequency of going out, a previous study reported that participants going out daily reported significantly fewer new complaints of musculoskeletal pain, sleep problems, urinary incontinence, and decline in activities of daily living [28]. In Japan, adjusted risks of incident mobility and IADL disabilities were significantly higher for those who went out “once a week or less often” compared with “once a day or more often” [26]. With regard to walking, a previous study reported that better maintenance of time spent walking over a decade predicted the maintenance of general cognitive performance [29]. An increase in time spent walking among sedentary adults is significantly associated with a lower risk of incident functional disability [30]. Our finding is consistent with those of the previous studies.

Cumulative LTCI costs significantly decrease with the frequency of going out and the time spent walking, after adjustment for baseline covariates, including physical function and depression. Cumulative LTCI costs for those who went out once a week or less were USD 700 higher than those who went out almost daily. Further, costs for those who walked for less than 30 min were USD 1100 higher than those who walked for more than 60 min. As far as we know, no previous research has investigated the relationship between physical activity and long-term care costs. On the other hand, many studies have investigated the effects of physical activity on medical expenditure [19,20,21,22]. One study [23], using a national dataset of Japanese adults, found that medical expenditures were significantly reduced in individuals who spent longer walking; the average per capita monthly medical cost for those who walked for less than 30 min was USD 26.1 higher than those who walked for more than 60 min. In our study, the difference in cumulative LTCI costs between “less than 30 min” and “more than 60 min” was USD 18.6, if we converted it into the monthly cost. This amount was equivalent to 0.71 times the medical cost in the previous study. Therefore, considering the LTCI costs, healthcare costs associated with physical inactivity will be 1.71 times as much as evaluated, based only on medical costs.

The Ministry of Health, Labour, and Welfare, Japan, established a 10-year plan that began in 2013, called “Health Japan 21”. The policies, ideas, and specific goals that form the basis of the plan are included in the “Basic Direction for Comprehensive Implementation of National Health Promotion”. The second term of Health Japan 21 aims to extend healthy life expectancy and reduce health disparities, establishing targets in 53 specific areas [31]. The target value for the number of steps is 7000 steps/day for men aged 65 years and older, and 6000 steps/day for women aged 65 years and older. A total of 6000 steps corresponds to approximately 60 min of walking when converted to time.

Based on our results, we can estimate the total cost reduction for LTCI in Japan if this target is achieved. Approximately 3500 million of the population was aged 65 years and older in the 2018 fiscal year [32]. When we extrapolate the results of this study to the whole country, there are approximately 11.6 million persons aged 65 years and older walking for 30 to 60 min. Those who walked for less than 30 min were excluded from this calculation because they were unlikely to achieve the target. The difference in cumulative LTCI costs between ”30 and 60 min” and “more than 60 min” was USD 300 per capita for 59 months. Based on this figure, the estimated total amount of cost reduction for LTCI is USD 34.8 billion, which translates to USD 7.1 billion, annually. It accounts for 7.0% of USD 101.5 billion, which was the total expense for long-term care in the 2018 fiscal year [33].

### 4.1. Strengths and Limitations

Our study is the first to report a difference in LTCI costs due to variations in PA, using a large sample from 11 municipalities. On the other hand, our study has several limitations. Firstly, our data do not reflect a nationally representative sample. Our findings need to be replicated in additional studies. Secondly, self-reported physical activity (PA) was also a limitation in this study, given the responses might have been misreported, and/or the definition of “going out” and “walk” might differ between respondents. Thirdly, 85% of participants were not certified as requiring long-term care in the follow-up period, and therefore, around 85% of cumulative LTCI costs are zero.

### 4.2. Implications

Despite these limitations, our study is the first to report a difference in LTCI costs due to variations in PA. To preserve older adults’ quality of life and manage health care costs, the promotion of PA in this age group is essential. Promoting PA requires knowledge of its associated correlates [34]. Most authors have adopted a social-ecological model, with growing attention to physical environmental correlates [35]. The physical environment in which people live, including walkability, accessibility (to services, shops, and public transport) and safety, influences their physical activities [36].

Considerable financial expenditure is required to change the physical environment. An evaluation of free public transport for older adults in the UK revealed that those with free passes not only traveled more often, but they were also more likely to walk further than those who did not receive free passes. The scheme costs approximately GBP 1 billion a year [37]. Municipalities provide public bus transport services in many rural areas of Japan. Amano et al. conducted a nationwide survey of municipalities in Japan and revealed that 98.4% of all local public bus routes operated under a deficit [38]. To assist local municipalities, the central government provided a total of USD 30 million a year in subsidies to compensate for the deficit in the 2018 fiscal year [39].

The cross-sector benefit of public transport services could justify a large sum of public bus service expenditures [40]. Cross-sector benefits are achievable in another sector of the economy due to expenditures in a particular sector. Some studies have shown that more accessible transit can relieve the demand and financial pressure on non-transportation social safety net programs [41]. The results of this study provide government agencies, health insurers, and health economists with accurate estimates of the increase in long-term care expenditures resulting from physical inactivity, which is essential information for calculating the cost-effectiveness of interventions to prevent and treat physical inactivity.

## 5. Conclusions

This prospective cohort study in Japan indicated that the frequency of going out and the time spent walking were significantly associated with the lower cost of LTCI for the first time. Based on these findings, and in addition to the impact of physical inactivity on medical expenditure reported in previous studies, we can now evaluate the expected effects of physical activity on the whole healthcare expenditure of persons aged 65 and over.

## Figures and Tables

**Table 1:** Baseline characteristics of the participants and cumulative long-term care insurance costs.

		% of Total Sample	Cumulative LTCI Cost Mean SD		*p*
Age	Mean ± SD	73.6 ± 5.9			
	65–69	29.8	0.4	4.6	0.049
	70–74	30.6	0.7	5.6	
	75–79	22.3	1.9	9.1	
	80–84	12.3	4.6	14.2	
	85+	5.1	8.2	18.4	
Sex	Female	53.3	2.0	9.9	0.001
	Male	46.7	1.5	7.8	
Marital status	Married	71.4	1.2	7.2	0.010
	Widowed	20.8	3.4	12.7	
	Divorced	3.4	1.3	7.0	
	Never married	2.2	3.2	13.4	
	Other	0.6	1.8	10.1	
	Missing	1.6	3.6	13.7	
Equivalent Income	40.0+	8.7	1.1	6.7	0.002
USD 1000	20.0–39.9	31.8	1.5	8.2	
	<20.0	41.6	1.8	8.9	
	Missing	17.9	2.6	10.9	
Years of education	≥13	17.8	1.3	7.1	0.008
	10–12	34.0	1.4	7.8	
	6–9	43.8	1.9	9.3	
	<6	2.0	6.0	18.1	
	Other	0.6	1.8	7.1	
	Missing	1.8	5.3	16.9	
Arthritis, fracture, osteoporosis	No	75.5	1.5	8.3	0.003
	Yes	15.2	2.7	10.9	
	Missing	9.4	2.3	10.1	
Heart disease	No	79.8	1.6	8.6	0.001
	Yes	10.8	2.3	10.0	
	Missing	9.4	2.3	10.1	
Stroke	No	89.5	1.7	8.8	0.001
	Yes	1.1	3.2	12.1	
	Missing	9.4	2.3	10.1	
Diabetes mellitus	No	64.0	1.9	9.3	0.001
	Yes	12.3	2.0	9.3	
	Missing	23.7	1.2	7.7	
Visual impairment	No	79.2	1.6	8.5	0.002
	Yes	11.5	2.6	10.8	
	Missing	9.4	2.3	10.1	
Hearing impairment	No	84.6	1.6	8.5	0.002
	Yes	6.0	3.2	12.1	
	Missing	9.4	2.3	10.1	
Urination disorders	No	84.6	1.6	8.5	0.002
	Yes	6.0	3.3	12.3	
	Missing	9.4	2.3	10.1	
Climbing stairs	Yes	59.4	1.2	7.4	0.006
	No	36.1	2.6	10.8	
	Missing	4.4	2.4	10.3	
Rising from chair	Yes	81.5	1.3	7.7	0.011
	No	14.3	4.0	13.4	
	Missing	4.2	2.6	11.3	
Self-rated health	Very good	12.0	0.9	6.1	0.008
	Good	69.1	1.5	8.2	
	Fair	15.7	3.1	11.7	
	Poor	2.1	4.4	13.8	
	Missing	1.1	3.6	15.3	
Geriatric Depression Scale	No	60.8	1.3	7.5	0.004
	mild	17.0	2.1	9.5	
	Severe	5.2	3.1	12.6	
	Missing	16.9	2.6	11.3	

Note: *p*-values were derived from ANOVA.

**Table 2:** Rate of those certified as requiring long-term care and mortality.

	*N*	Certified as Requiring Long-Term Care			Mortality		
		*n*	1000 Person-Years	Rate	*n*	1000 Person-Years	Rate
Frequency of going out							
Almost every day	20,396	2301	94.7	24.3	1249	98.2	12.7
Two or three times per week	10,606	1787	48.0	37.3	741	50.9	14.6
Once a week or less	5603	1246	24.3	51.2	610	26.5	23.0
Missing	2270	403	10.2	39.5	179	10.8	16.5
Time spent walking							
≥60 min	11,817	1088	55.2	19.7	638	57.0	11.2
30–59 min	12,833	1588	59.0	26.9	796	61.7	12.9
<30 min	11,768	2043	52.0	39.3	1155	55.8	20.7
Missing	2457	451	10.9	41.2	190	11.7	16.2

Note: *N* indicates the number of participants in each category, *n* indicates the number of participants certified as requiring long-term care or who died during the follow-up, and the rate is the number of functional limitations or deaths per 1000 person-years.

**Table 3:** Differences in cumulative cost of long-term care insurance services by frequency of going out.

		GLM		OLS		GLM with MI *		OLS with MI *	
	*N*	Coef. (95%CI)	*p*	Coef. (95%CI)	*p*	Coef. (95%CI)	*p*	Coef. (95%CI)	*p*
Frequency of going out									
Almost every day	20,421	Ref.		Ref.		Ref.		Ref.	
Two or three times per week	10,614	0.15 (0.01 to 0.30)	0.040	0.15 (0.05 to 0.25)	0.003	0.14 (−0.03 to 0.28)	0.055	0.16 (0.07 to 0.26)	0.001
Once a week or less	5612	0.24 (0.09 to 0.39)	0.001	0.26 (0.12 to 0.41)	<0.001	0.24 (0.10 to 0.39)	0.001	0.25 (0.12 to 0.38)	<0.001

Unit: USD 1000. The results were controlled by sex, age, disease and/or impairment, years of education, equivalent income, marital status, depression, and self-rated health at baseline. Missing values in the covariates were dummy coded and included as the “Missing” category. * Multiple imputations by chained equations were performed using sex, age, disease and/or impairment, years of education, equivalent income, marital status, depression, and self-rated health at baseline (m = 20). The sample size of the complete data was 36605.

**Table 4:** Differences in cumulative cost of long-term care insurance services by time spent walking.

		GLM		OLS		GLM with MI *		OLS with MI *	
	*N*	Coef. (95%CI)	*p*	Coef. (95%CI)	*p*	Coef. (95%CI)	*p*	Coef. (95%CI)	*p*
≥60 min	11,294	Ref.		Ref.		Ref.		Ref.	
30–59 min	12,256	0.13 (−0.03 to 0.29)	0.118	0.13 (−0.10 to 0.33)	0.007	0.12 (−0.03 to 0.28)	0.123	0.14 (0.03 to 0.24)	0.010
<30 min	11,247	0.43 (0.27 to 0.59)	<0.001	0.33 (0.42 to 0.87)	<0.001	0.43 (0.27 to 0.59)	<0.001	0.32 (0.22 to 0.43)	<0.001

Unit: USD 1000. The results were controlled by sex, age, disease and/or impairment, years of education, equivalent income, marital status, depression, and self-rated health at baseline. Missing values in the covariates were dummy coded and included as the “Missing” category. * Multiple imputations by chained equations were performed using sex, age, disease and/or impairment, years of education, equivalent income, marital status, depression, and self-rated health at baseline (m = 20). The sample size of the complete data was 36,418.

**Table 5:** Adjusted mean of estimated cumulative cost of long-term care insurance services by physical activity.

		GLM	GLM with MI *
	*N*	Estimate (95%CI)	Estimate (95%CI)
Frequency of going out			
Almost everyday	20,421	2.3 (1.7 to 3.1)	2.4 (1.7 to 3.1)
Two or three times per week	10,614	2.7 (2.0 to 3.7)	2.7 (1.9 to 3.5)
Once a week or less often	5612	3.0 (2.2 to 4.1)	3.0 (2.1 to 3.9)
Time spent walking			
≥60 min	11,294	2.1 (1.4 to 3.0)	2.1 (1.5 to 2.7)
30–59 min	12,256	2.4 (1.7 to 3.4)	2.3 (1.7 to 3.0)
<30 min	11,247	3.2 (2.2 to 4.6)	3.2 (2.3 to 4.1)

Unit: USD 1000. The results were controlled by sex, age, disease and/or impairment, years of education, equivalent income, marital status, depression, and self-rated health at baseline. Missing values in the control variables were included as the “Missing” category. * Multiple imputations by chained equations were performed using sex, age, disease and/or impairment, years of education, equivalent income, marital status, depression, and self-rated health at baseline (m = 20).

## Data Availability

Data are not open for public due to ethical concerns. Data are from the JAGES study whose authors may be contacted at data management committee: dataadmin@jages.net. The data set has ethical or legal restrictions because it includes human participants.
